# Response to commentaries by Schmidt and Kaplan, Penhune, Hickok and Theofanopoulou on “Beat-based dancing to music has evolutionary foundations in advanced vocal learning.”

**DOI:** 10.1186/s12868-024-00853-4

**Published:** 2024-11-06

**Authors:** Aniruddh D. Patel

**Affiliations:** Dept. of Psychology, Tufts University & Program in Brain, Mind, and Consciousness, Canadian Institute for Advanced Research, 490 Boston Ave, 02155 Medford, MA USA

**Keywords:** Rhythm, Evolution, Brain, Dance, Vocal learning, Parietal cortex, Synchrony, Speech

## Abstract

Each commentary on my article raises important points and new ideas for research on rhythmic processing in humans and other species. Here I respond to points concerning the role of social factors in the ontogeny of beat synchronization, the neural connectivity underlying beat synchronization, the evolution of this connectivity, and the mechanisms by which evolutionary changes in the strength of one white matter tract (driven by natural selection) can have knock-on effects on the structure of an adjacent tract.

## Main text

### Schmidt and Kaplan

Schmidt and Kaplan [1] (henceforth S&K) point out that even though beat perception and synchronization (BPS) might develop without formal training in pet parrots, it is important to consider the role of reinforcement in the emergence of this behavior. S&K note that social reinforcement by humans (e.g., via attention or joint dancing) might be a significant influence in the development of parrot BPS. They note that such reinforcement would constitute reward for parrots, who are highly social animals. I agree and also concur with S&K’s related point that “this is not unlike the way human children learn to move to music: gradually, and with social reward over many years.” In the case of humans, my hunch (based on research with infants [2, 3]) is that a predisposition to move rhythmically to beat-based music is widespread, and that this initial spark can be strongly amplified by social reinforcement. To determine if something similar holds for pet parrots, it would be interesting to conduct a survey of parrot owners about the ontogeny of such movements, modeled on a recent survey-based study about infant rhythmic movement to music [3].

Sparked by S&K’s ideas, I contacted Irene Pepperberg, whose cognitive research with Alex the African grey parrot is well known in psychology [4, 5]. In a 2009 study Pepperberg and colleagues showed that Alex synchronized his head bobs to the beat of novel rhythmic music [6]. In email correspondence, Dr. Pepperberg told me she acquired Alex from a pet store when he was about one year old and never formally trained him to move to a beat. She mentioned Alex was tested for BPS at age 31 and had not participated in any music cognition experiments previously. Dr. Pepperberg also noted that Alex’s BPS movements were relatively small compared to those of Snowball the cockatoo. Readers can compare Alex’s modest head bobs to Snowball’s exuberant head bobs and foot-stomps via the supplementary videos published in [6] and [7].

As described in [7], soon after being acquired at a pet show at age 6 Snowball lived with a family who noticed his rhythmic head bobs to music and began dancing with him. The family cared for him for four years before relinquishing him to the bird shelter where his BPS experiment took place at age 11. Thus, in line with S&K’s suggestion, it seems plausible that social reinforcement led Snowball from modest head bobs to larger rhythmic movements to music. One wonders if Alex would have gone through a similar trajectory if Dr. Pepperberg had socially reinforced his rhythmic responses to music.

In an interesting paragraph about whether nonhuman animals get pleasure from human music, S&K say that like humans, “Parrots might also experience pleasure in responding to the musical beat, and this might be tied to their behavioral ecology, to the accuracy of their own sensorimotor predictions, or to social reward in the form of approval from their handlers. This raises an important question: does Snowball dance by himself, even when no one is watching?” I am fascinated by S&K’s idea that rhythmic movement to music in pet parrots involves *multiple* sources of pleasure unrelated to food rewards, and I am pleased to say that data analysis is currently underway from a study examining if Snowball dances when alone.

### Penhune

Penhune’s commentary [8] raises interesting ideas about the proposed cortical beat processing pathway in my paper. That pathway links cortical auditory and dorsal premotor regions via the angular gyrus in the inferior parietal cortex (see Fig. 4A of my target article [9]). My paper focused on the evolutionary strengthening of connections between auditory and inferior parietal cortices (red line in Fig. 4A of [9], corresponding to the temporo-parietal branch of the superior longitudinal fasciculus [SLF-tp] in Fig. 4B of [9], a pathway also known as the posterior segment of the arcuate fasciculus). Penhune calls attention to the other part of this pathway, linking inferior parietal to dorsal premotor cortices (orange line in Fig. 4A of [9], corresponding to the second branch of the superior longitudinal fasciculus [SLF II] in Fig. 4B of [9]). Penhune notes that comparative neuroanatomical studies by Petrides and colleagues suggest that evolutionary changes in SLF II in the primate brain were important for human cognition, with recent work by this group finding that white matter integrity in SLF II predicts specific aspects of second-language learning. Interestingly, SLF II is involved in a variety of cognitive abilities: prior work indicates it plays a role in visuomotor processing and working memory, among other functions [10]. This raises the question of whether beat processing abilities correlate with any linguistic or non-musical cognitive abilities that depend on SLF II, a question amenable to empirical research. Should this prove to be the case, it would be interesting to see if beat-based rhythmic training enhances those abilities in children, during a time when parieto-frontal white matter tracts are still myelinating. Such work would enrich the growing body of studies finding links between beat processing and other cognitive functions, including language (e.g., [11, 12]), a line of research with implications for language acquisition and with clinical significance.

### Hickok

Hickok [13] offers an interesting alternative to my proposal. My paper suggests that the evolution of one of two neural pathways in a dual-stream system for vocal control fortuitously led to a capacity for BPS in parrots and human ancestors. In contrast, Hickok proposes that a capacity for BPS is byproduct of evolving a neural system that *coordinates* the activity of these two pathways. He suggests “beat synchronization is a simple version of what’s required for speech coordination.”

At first this proposal seems puzzling since Hickok and I agree that ordinary speech does not have beat-based rhythms [14]. Hickok suggests, however, that speech has quasi-periodic rhythms with some degree of predictability. Specifically, he suggests that speech production planning involves “an auditory-based representation of the quasi-rhythmic target, which is used as a temporal reference to synchronize articulatory planning of separately controlled effectors. Once the system has the ability to generate an auditory-based target rhythm and, crucially, to wire it up to motor systems for the purpose of synchronizing to it, an externally provided and *predictable* rhythm would then function as a (simpler) target for motor synchronization, i.e., BPS emerges for free.”

I am intrigued by Hickock’s hypothesis and hope it leads to strong predictions that are tested in future work. Hickok notes one such prediction, namely that “parrots should exhibit vocal abilities that require some degree of temporally precise coordination between syringeal and suprasyringeal effectors.” In terms of human BPS research, another prediction would be that individuals with difficulty coordinating laryngeal and supralaryngeal movements during speech would have difficulty with BPS. This could be tested by studying nonvocal beat synchronization (e.g., tapping) in individuals with apraxia of speech involving laryngeal-supralaryngeal coordination problems.

One concern I have with Hickok’s hypothesis is the idea that BPS is a simpler version of a neural coordination process involved in speech. How does this view account for the existence of “beat-deaf” individuals who have great difficulty synchronizing to and/or perceiving a musical beat, yet who do not suffer from any obvious speech problems [15, 16]? Another concern is the notion that beat-based rhythms are simple versions of the auditory-based “quasi-rhythmic” patterns involved in speech planning. Current research in music cognition suggests that beat-based rhythmic processing dissociates from more complex temporal pattern processing behaviorally and neurally [17, 18], which seems inconsistent with Hickok’s view.

Hickok correctly notes that a key challenge for my proposal is explaining why the evolution of a laryngeal pitch control pathway in humans would influence anatomically distinct pathways involved in beat processing. My paper conjectures that gene regulation changes involved in the evolution or strengthening of the laryngeal pitch-control pathway (e.g., via increases the number, diameter, and/or myelination of its axons) fortuitously strengthened connections in an auditory-to-inferior parietal pathway involved in beat processing (red line in my Fig. 4A, connecting auditory cortex and angular gyrus via the temporo-parietal branch of the superior longitudinal fasciculus [SLF-tp], cf. Figure 4B). However, many details and implications of this idea remain to be explored. For example, twin studies show that the structure of SLF-tp has a substantial genetic influence [19], but which genetic variants influence the strength of this tract, and do these SLF-tp variants overlap with other genetic variants influencing the strength of the laryngeal pitch control pathway? More generally, can a gene regulation change that increases the strength of one tract have a knock-on effect on a neighboring tract? If so, are any parts of the laryngeal pitch control pathway in close physical proximity to SLF-tp in early brain development, when long-distance white matter pathways are forming [20]?

Relevant to this last question about anatomical proximity, based on fMRI research it seems likely that the laryngeal pitch control system includes an indirect connection between auditory and dorsal premotor regions via area Spt [21, 22] (see Fig. 4A of my paper for approximate location of Spt). Spt overlaps with part of supramarginal gyrus (SMG), the gyrus surrounding the superior end of the Sylvian fissure. SMG and angular gyrus (AG) are adjacent regions of the inferior parietal cortex [23], a part of the brain which shows a disproportionate amount of postnatal expansion in human brain development compared to other cortical regions, and which expanded dramatically in human evolution [24]. Thus auditory-Spt and auditory-AG connections may be in closer proximity in early brain development (and in human ancestors) than they are in modern adult brains. If true, this could help explain why gene-regulation changes influencing the strength of the former tract would fortuitously influence the strength of the latter tract. These issues await future research using modern methods which are elucidating the genetic and developmental mechanisms underlying the evolution of cortical circuits [25, 26].

### Theofanpoulou

In a thought-provoking commentary [33], Theofanpoulou discusses evidence relevant to my target article’s proposal that gene regulation changes influencing the structure of one white matter tract could fortuitously influence the structure of a neighboring tract. She helpfully gives examples of experimental studies in small animals in which gene regulation changes in a target neuron have effects on neural structure or gene regulation in adjacent neurons, but notes that current research provides limited evidence for this “spread” of gene expression effects between neighboring neurons. I fully agree that more information on mechanisms is needed to substantiate my proposal, and suggest that one promising avenue of research is to examine genetic correlations between neighboring white matter tracts.

There have been recent advances in studying the genetics of long-distance white matter tracts in the human brain, made by leveraging large-scale imaging and genomics databases such as the UK Biobank [e.g., 27, 28, 29]. These studies have discovered genetic variants associated with individual differences in the strength of several white matter tracts and have found genetic correlations between these sets of variants and sets of genetic variants associated with cognitive traits. These studies also find that genes associated with the strength of white matter tracts are associated with a range of neurodevelopmental processes, including axon development and myelination. Using methods developed in these studies, one could measure whether neighboring white matter tracts have greater genetic correlation than more distant tracts. If so, this would be in line with my proposal that evolutionary strengthening of one white matter tract could have knock-on effects on a nearby tract, because the tracts are not under independent genetic control. In particular, it would be interesting to determine if some of the genetic variants shared by neighboring tracts are part of overlapping gene regulatory networks. If so, such variants could lead to spatial correlations in gene expression in nearby tracts, analogous to the spatial correlations in gene expression seen in nearby cortical gray matter regions [30]. Such findings would provide a mechanism by which gene regulation changes in one tract (driven by natural selection to strengthen that tract) could fortuitously influence the structure of a neighboring tract.

Theofanpoulou’s commentary also proposes an intriguing hypothesis concerning relations between neural populations in motor cortex controlling vocal effectors (such as the larynx) and nonvocal effectors such as hand or leg muscles. Surveying a range of relevant studies in humans and other animals, she suggests that *overlapping* (in addition to adjacent) neural populations in primary motor cortex (M1) can be involved in vocal and nonvocal movements. In line with this view, Theofanpoulou points to her ongoing fMRI work with colleagues showing neural overlap in M1 regions involved in speech-related laryngeal movements and in nonvocal rhythmic movements used in dance. She also suggests methods to test her hypothesis with finer-grain spatial and temoral resolution, including using Neuropixel electrodes to measure activity in different layers of M1 as participants speak or engage in rhythmic nonvocal movements. If Theofanpoulou’s hypothesis is borne out, it could help explain why associations have been found between atypical rhythmic processing and a range of speech and language deficits [31], and would raise the intriguing and counterintuitive possibility that dance-based interventions could enhance certain aspects of speech processing in children with speech and language disorders. Should this prove to be true, it would be a fascinating example of how music-based activities can benefit other brain functions [32].

## Conclusions

The five scholars who commented on my paper bring expertise in the cognitive neuroscience of animal behavior (Schmidt & Kaplan) [1], human music (Penhune) [8], speech (Hickok) [13], and vocal learning (Theofanpoulou) [33] to bear on the ideas presented in my article. Their points and critiques illustrate how dialogues across traditional disciplinary boundaries can advance evolutionary research on musicality. Due to space limitations my response focuses on a subset of the points that they raised, concerning social factors in the ontogeny of beat synchronization, the neural connectivity underlying beat synchronization, the evolution of this connectivity, and the mechanisms by which evolutionary changes in the strength of one white matter tract can have knock-on effects on the structure of an adjacent tract.

## Data Availability

Not applicable.

## References

[1] Schmidt MF, Kaplan M. Toward a comparative understanding of beat perception and synchronization. BMC Neurosci. 25, 2024. 10.1186/s12868-024-00852-5.

[2] Zentner M, Eerola T. Rhythmic engagement with music in infancy. Proc Natl Acad Sci. 2010;107:5768–73. 20231438 10.1073/pnas.1000121107 PMC2851927

[3] Kim M, Schachner A. The origins of dance: characterizing the development of infants’ earliest dance behavior. Dev Psychol. 2023;59:691–706. 36095245 10.1037/dev0001436

[4] Pepperberg IM. The Alex studies: cognitive and communicative abilities of grey parrots. Cambridge, MA: Harvard University Press; 2002.

[5] Pepperberg IM. Symbolic communication in the Grey parrot. In: Vonk J, Shackelford T, editors. Oxford Handbook of comparative evolutionary psychology. NY: Oxford University Press; 2012. pp. 297–319.

[6] Schachner A, Brady TF, Pepperberg IM, Hauser MD. Spontaneous motor entrainment to music in multiple vocal mimicking species. Curr Biol. 2009;19:831–6. 19409786 10.1016/j.cub.2009.03.061

[7] Patel AD, Iversen JR, Bregman MR, Schulz I. Experimental evidence for synchronization to a musical beat in a nonhuman animal. Curr Biol. 2009;19:827–30. 19409790 10.1016/j.cub.2009.03.038

[8] Penhune VB. How different are the differences? A commentary on the paper “Beat-based dancing to music has evolutionary foundations in vocal learning” BMC Neurosci. 25, 2024. 10.1186/s12868-024-00850-7 10.1186/s12868-024-00843-6 PMC11539772 39506663

[9] Patel AD. Beat-based dancing to music has evolutionary foundations in advanced vocal learning. BMC Neurosci. 25, 2024. 10.1186/s12868-024-00843-6 10.1186/s12868-024-00843-6 PMC11539772 39506663

[10] Nakajima R, Kinoshita M, Shinohara H, Nakada M. The superior longitudinal fascicle: reconsidering the fronto-parietal neural network based on anatomy and function. Brain Imaging Behav. 2020;14:2817–30. 31468374 10.1007/s11682-019-00187-4

[11] Woodruff Carr K, White-Schwoch T, Tierney AT, Strait DL, Kraus N. Beat synchronization predicts neural speech encoding and reading readiness in preschoolers. Proceedings of the National Academy of Sciences. 2014;111:14559-64. 10.1073/pnas.1406219111 PMC4210020 25246562

[12] Nayak S, Coleman PL, Ladányi E, Nitin R, Gustavson DE, Fisher SE, Magne CL, Gordon RL. The musical abilities, pleiotropy, language, and environment (MAPLE) framework for understanding musicality-language links across the lifespan. Neurobiology of Language. 2022;3(4):615–64. 10.1162/nol_a_00079 PMC9893227 36742012

[13] Hickok G. The “coordination conjecture” as an alternative to Patel’s fortuitous enhancement hypothesis for the relation between vocal learning and beat-based dancing. BMC Neurosci. 25, 2024. 10.1186/s12868-024-00868-x

[14] Patel AD. Music, language, and the brain. NY: Oxford University Press; 2008.

[15] Phillips-Silver J, Toiviainen P, Gosselin N, Piché O, Nozaradan S, Palmer C, Peretz I. Born to dance but beat deaf: a new form of congenital amusia. Neuropsychologia. 2011;49(5):961–9. 21316375 10.1016/j.neuropsychologia.2011.02.002

[16] Tranchant P, Lagrois MÉ, Bellemare A, Schultz BG, Peretz I. Co-occurrence of deficits in beat perception and synchronization supports implication of motor system in beat perception. Music Sci. 2021;4:2059204321991713.

[17] Fiveash A, Bella SD, Bigand E, Gordon RL, Tillmann B. You got rhythm, or more: The multidimensionality of rhythmic abilities. Attention, Perception, & Psychophysics. 2022;84(4):1370-92. 10.3758/s13414-022-02487-2 PMC9614186 35437703

[18] Bouwer FL, Fahrenfort JJ, Millard SK, Kloosterman NA, Slagter HA. A silent disco: Differential effects of beat-based and pattern-based temporal expectations on persistent entrainment of low-frequency neural oscillations. J Cogn Neurosci. 2023;35(6):990–1020. 36951583 10.1162/jocn_a_01985

[19] Budisavljevic S, Dell’Acqua F, Rijsdijk FV, Kane F, Picchioni M, McGuire P, Toulopoulou T, Georgiades A, Kalidindi S, Kravariti E, Murray RM. Age-related differences and heritability of the perisylvian language networks. J Neurosci. 2015;35:12625–34. 26377454 10.1523/JNEUROSCI.1255-14.2015 PMC4571601

[20] Harris WA. Zero to birth: how the human brain is built. Princeton: Princeton University Press; 2022.

[21] Hickok G, Buchsbaum B, Humphries C, Muftuler T. Auditory–motor interaction revealed by fMRI: speech, music, and working memory in area Spt. J Cogn Neurosci. 2003;15(5):673–82. 12965041 10.1162/089892903322307393

[22] Hickok G. A cortical circuit for voluntary laryngeal control: implications for the evolution language. Psychon Bull Rev. 2017;24:56–63. 27368637 10.3758/s13423-016-1100-z PMC5652042

[23] Wild HM, Heckemann RA, Studholme C, Hammers A. Gyri of the human parietal lobe: volumes, spatial extents, automatic labelling, and probabilistic atlases. PLoS ONE. 2017;12(8):e0180866. 28846692 10.1371/journal.pone.0180866 PMC5573296

[24] Hill J, Inder T, Neil J, Dierker D, Harwell J, Van Essen D. Similar patterns of cortical expansion during human development and evolution. Proc Natl Acad Sci. 2010;107(29):13135–40. 20624964 10.1073/pnas.1001229107 PMC2919958

[25] Zhou Y, Song H, Ming GL. Genetics of human brain development. Nat Rev Genet. 2023. 10.1038/s41576-023-00626-5. 37507490 10.1038/s41576-023-00626-5 PMC10926850

[26] Vanderhaeghen P, Polleux F. Developmental mechanisms underlying the evolution of human cortical circuits. Nat Rev Neurosci. 2023;24(4):213–32. 36792753 10.1038/s41583-023-00675-z PMC10064077

[27] Zhao B, Li T, Yang Y, Wang X, Luo T, Shan Y et al. Common genetic variation influencing human white matter microstructure. Science. 2021;372(6548):eabf3736. 10.1126/science.abf3736 PMC8370718 34140357

[28] Sha Z, Schijven D, Fisher SE, Francks C. Genetic architecture of the white matter connectome of the human brain. Science advances. 2023;9(7):eadd2870 10.1126/sciadv.add2870 PMC9937579 36800424

[29] Wainberg M, Forde NJ, Mansour S, Kerrebijn I, Medland SE, Hawco C, Tripathy SJ. Genetic architecture of the structural connectome. Nature Communications. 2024;15(1):1–20 10.1038/s41467-024-46023-2 PMC10912129 38438384

[30] Kong XZ, Tzourio-Mazoyer N, Joliot M, Fedorenko E, Liu J, Fisher SE, Francks C. Gene expression correlates of the cortical network underlying sentence processing. Neurobiology of Language. 2020;1(1):77–103 10.1162/nol_a_00004 PMC9923707 36794006

[31] Ladányi E, Persici V, Fiveash A, Tillmann B, Gordon RL. Is atypical rhythm a risk factor for developmental speech and language disorders?. Wiley Interdisciplinary Reviews: Cognitive Science. 2020;11(5):e1528. 10.1002/wcs.1528 PMC7415602 32244259

[32] Fleming, R. (Ed.) Music and mind: harnessing the arts for health and wellness. NY: Penguin Random House; 2024.

[33] Theofanopoulou, C. Tapping into the vocal learning and rhythmic synchronization hypothesis BMC Neurosci. 25, 2024. 10.1186/s12868-024-00863-2. 10.1186/s12868-024-00863-2 PMC11539701 39506690

